# Identification of Candidate Genes Associated with Pulp Color by Transcriptomic Analysis of ‘Huaxiu’ Plum (*Prunus salicina* Lindl.) during Fruit-Ripening

**DOI:** 10.3390/cimb44120434

**Published:** 2022-12-15

**Authors:** Gang Wang, Wenxin Weng, Zhanhui Jia, Jiyu Zhang, Tao Wang, Jiping Xuan

**Affiliations:** Jiangsu Key Laboratory for the Research and Utilization of Plant Resources, Institute of Botany, Jiangsu Province and Chinese Academy of Sciences, Nanjing 210014, China

**Keywords:** *Prunus salicina*, pulp color, transcriptome, RNA-Seq, DGE, anthocyanin and flavonoid biosynthesis

## Abstract

The plum (*Prunus salicina* Lindl.) is one of the traditional and economically important stone fruit trees in China. Anthocyanins are important pigments in plums. However, little is known about the molecular mechanisms underlying anthocyanin accumulation in plum fruits, which has hindered research on the molecular mechanism of its utilization. Our research shows that the chlorophyll content was gradually decreased and the contents of anthocyanin and flavonoid increased during the coloring process of the pulp in ‘Huaxiu’ plums (*P. salicina*). Then, the RNA-Seq technique was used to analyze the transcriptome of pulp color changes with three different stages (yellow, orange, and red) in the ‘Huaxiu’ plum (*P. salicina*). A total of 57,119 unigenes with a mean length of 953 bp were generated, and 61.6% of them were annotated to public databases. The Gene Ontology (GO) database assigned 21,438 unigenes with biological process, cellular components, and molecular function. In addition, 32,146 unigenes were clustered into 25 categories for functional classification by the COG database, and 7595 unigenes were mapped to 128 KEGG pathways by the KEGG pathway database. Of these, 1095 (YS-versus-OS), 4947 (YS-versus-RS), and 3414 (OS-versus-RS) genes were significantly expressed differentially between two coloration stages. The GO and KEGG pathway enrichment analysis revealed that 20 and 1 differentially expressed genes (DEG) are involved in flavonoid biosynthesis and anthocyanin biosynthesis, respectively. Finally, we mainly identified three structural genes as candidate genes. The transcriptome information in this study provide a basis for further studies of pulp colors in plum and contribute to our understanding of the molecular mechanisms underlying anthocyanin biosynthesis in pulp.

## 1. Introduction

The plum (*Prunus salicina* L.) is an economically important stone fruit tree in China. The fruit can be eaten fresh or processed into many value-added products, such as juice, fermented wine, and preserves [1]. When the fruit matures, the pulp color can be green, yellow–green, yellow, red, or purple based on the difference cultivar of the plum. The fruit color is mainly determined by the level of anthocyanin accumulation [2]. Anthocyanins have been extensively studied, not only because they result in the production of red, purple, and black pigments, but also in the contexts of their diverse roles in UV protection and pathogen defense, as well as their nutritional value in the human diet [3].

Anthocyanins are formed by the combination of anthocyanidin and one or more carbohydrates through glycosides bonds which then stably exist in plant cells, which make the fruit appear bright colors [4]. Anthocyanins are synthesized through the flavonoid pathway which has been well-studied in many plants. Many structural genes of the flavonoid pathway have been isolated and identified as the critical gene for anthocyanin biosynthesis in plants, such as chalcone synthase (CHS), chalcone isomerase (CHI), flavonoid 3′-hydroxylase (F3′H), anthocyanidin synthase (ANS), and UDP-glucose: flavonoid 3-O-glucosyltransferase (UFGT) [4,5,6,7]. In addition, transcription factors play important roles in modulating anthocyanin biosynthetic pathway activity and color changes, including MYB proteins, basic helix–loop–helix (bHLH) proteins, and WD40 proteins [8]. These regulators form an MYB–bHLH–WD40 (MBW) complex that binds to promoters and regulates the expression of structural genes of the anthocyanin biosynthetic pathway [9,10]. The role of MBW in anthocyanin biosynthesis has been elucidated in fruit trees such as apple [11], pears [12,13], grape [14], strawberry [15], Chinese bayberry [16], kiwifruit [17], litchi [18], blueberry [19], sweet cherry [20] peach [21,22], plum [1], and fig [23]. In addition, other regulatory factors also affect anthocyanin biosynthesis via interaction with the MBW complex or by modulating the transcription of structural genes directly [1]. A SQUAMOSA promoter-binding protein-like (SPL) transcription factor (*PpSPL1*) has been shown to inhibit the expression of anthocyanin biosynthetic genes and negatively regulate anthocyanin accumulation through the destabilization of MBW [24]. MADS-box and NAC transcription factors also are reported to be involved in the regulation of anthocyanin accumulation [25,26].

In recent years, RNA sequencing (RNA-Seq) transcriptome analysis has been extensively used for the identification of functional genes in fruit trees, including plum. The genome of Chinese plum ‘Sanyueli’ and the chloroplast genome sequence of ‘Wushan plum’ (*P. salicina*) have been published [27,28]. The transcriptome analysis of ‘Siyueli’-pollinated and ‘Yinhongli’-pollinated fruits revealed 2762 and 1018 differentially expressed genes (DEGs) involved in the response to different pollen sources [29]. Recently reported are the fruit skin transcriptome assembly of ‘Angeleno’ and ‘Lamoon’ Japanese plum cultivars with different skin color [30], and the transcriptomic changes during fruit-ripening in the red-fleshed plum cultivar ‘Furongli’ (*P. salicina*) [1]. During the ripening and development of plum fruits, chlorophyll degradation, the content of organic acids decreases, while anthocyanins accumulate rapidly in the colored varieties, and this phenomenon is especially obvious in the late stage of plum fruit-ripening [31]. In this study, as a first step towards understanding gene expression during fruit-ripening in plum, the three mature stages of ‘Huaxiu’ plum fruits with pulp colors of yellow, orange, and red were collected for transcriptome sequencing. This study presents the results of a comprehensive analysis of transcriptome data from fruits at three representative developmental stages. The results will help us to understand the gene expression difference and explore the molecular mechanisms of the biosynthetic pathways in secondary metabolites in plum.

## 2. Materials and Methods

### 2.1. Plant Materials

Fruits of ‘Huaxiu’ plum (*P. salicina*) were collected from 6-year-old field-grown trees in an orchard in Donghai County, Jiangsu Province, China. In July 2007, a natural-bud mutation for early maturing fruit of ‘Qiuji’ was found by a farmer and named as ‘Huaxiu’. According to our observations, ‘Huaxiu’ characteristics are mid-early ripening (fruits mature in late-July in this area, about 108 days after flowering), where the skin is dark purple and the pulp orange–red. These mature fruit samples were divided into three different stages based on pulp color and termed the ‘Yellow stage’ (YS), ‘Orange stage’ (OS), ‘Red stage’ (RS), respectively.

During the fruit ripening periods, three biological replicates were collected per sample, each with 20 fruits randomly collected from two trees in order to decrease background variation. Then, three pulps were peeled and sliced into appropriate pieces after measuring the weight and diameter, and then immediately frozen in liquid nitrogen for the determination of chlorophyll, carotenoid, anthocyanins, and flavonoids. Two biological replicated samples were used at each stage for transcriptome sequencing due to the background and limited yield of fruits.

### 2.2. Total Anthocyanin, Flavonoid, and Chlorophyll Measurements

Total anthocyanin levels were determined according to the HPLC system [32]. Approximately 1.5 g of the sample was ground to a fine powder in liquid nitrogen and extracted with a 1% HCl-methanol solution at 4 °C for 24 h to obtain the extract by filtration. Then, 20 µL of the samples were injected into a C18 column (Agilent Zorbax Eclipse SB-C18, 4.6 mm × 100 mm, 1.8 µm, Santa Clara, CA, USA). The binary solvent system was 5% formic acid in water as mobile phase A and methanol as mobile phase B. The gradient elution was 20% B at 0–30 min, 40% B at 30–40 min, 100% B at 40 min. The flow rate was kept at 10 mL/min, and the column temperature was maintained at 40 °C. The chlorophyll and carotenoids levels were measured according to the absorbance at 663 nm, 645 nm, and 470 nm (A_663_, A_645_, and A_470_) [33]. The flavonoid levels were measured as previously described [34]. Each sample comprised three biological replicates and the analysis represented the results of three independent experiments.

### 2.3. RNA Extraction, cDNA Library Construction, and Sequencing

Total RNA from approximately 100 mg of frozen fruits of YS, OS, and RS was extracted using the TRIzol 1 Reagent (Invitrogen, Waltham, MA, USA) according to manufacturer’s protocol. Genomic DNA was eliminated by using RNase-free DNase I (TaKaRa, Dalian, China) and then RNA integrity and purity was confirmed on 1% agarose gels and NanoDrop™ 2000 spectrophotometer (NanoDrop Technologies, Wilmington, DE, USA). All RNA extracts showed a 260/280 nm ratio from 1.9 to 2.2. Approximately 25 μg total RNA of no less than 600 ng/μL concentration was used for the cDNA library construction. The steps of the cDNA library construction and the subsequent sequencing using an Illumina HiSeq™ 4000 (San Diego, CA, USA) were performed by staff at Gene Denovo Biotechnology Co. (Guangzhou, China).

### 2.4. De Novo Assembly and Functional Annotation

First, the raw data were filtered to remove reads with unknown sequencing ‘N’ and low-quality reads with more than 50% bases with a quality value ≤5. Then, mixing the clean reads from the three samples, the de novo assembly was performed using the assembly software Trinity (Trinityrnaseq_r2013_08_14 version) to obtain transcript sequences, and the longest transcript in each gene was taken as a single gene cluster (unigene) [35]. To annotate unigenes sequences of ‘Huaxiu’ plums, Blastx search (E-value < 10^−5^) was used to search against the NCBI nonredundant protein database [36] (NR, http://www.ncbi.nlm.nih.gov/refseq/about/nonredundantproteins, last accessed on 23 August 2022), the SwissProt protein database (http://www.expasy.ch/sprot, last accessed on 24 August 2022), the Kyoto Encyclopedia of Genes and Genomes database [37] (KEGG, http://www.genome.jp/kegg, last accessed on 24 August 2022), and the Clusters of Orthologous Groups of proteins database [38] (COG, http://www.ncbi.nlm.nih.gov/COG, last accessed on 24 August 2022). These annotation databases were used for comparisons with homologous genes. GO function classification was performed using Blast2GO [39] (http://www.blast2go.com/b2ghome, last accessed on 14 September 2022) with E ≤ 10^−5^, the distribution of the GO functional classifications of the unigenes was plotted using WEGO software [40], which allowed categorization into three different GO terms, including molecular function, cellular component, and biological process. The KEGG pathway annotation was performed using the Blast software against the KEGG database [41].

### 2.5. Expression Analysis

After assembly, unigene expression levels were quantified using fragments per kilo base of transcript per million mapped reads (FPKM). FPKM values were calculated using RSEM (RNA-Seq by Expectation Maximization) [42]. Differentially expressed genes (DEGs) analysis of two samples was performed using the DEG Seq R package [43]. The differentially expressed unigenes between two samples were screened using a false discovery rate (FDR), which is used to determine *p*-value thresholds in multiple testing [44]. The significance of the DEGs were determined based on a threshold of FDR <0.05 and absolute log2 fold changes ≥1 in a comparison as significant DEGs. GO enrichment analysis provided all GO terms that significantly enriched in DEGs compared to the genome background and filtered the DEGs that corresponded to biological functions. KEGG pathway enrichment analysis identified significantly enriched metabolic pathways or signal transduction pathways in DEGs compared with the whole genome background. DEGs were subsequently mapped to the database for the GO and KEGG pathway enrichment analysis with the *p*-value < 0.05 using the software GOatools [45] (https://github.com/tanghaibao/GOatools, last accessed on 16 September 2022).

Twelve DEGs involved in flavonoid biosynthesis were selected for validation by real-time quantitative RT-PCR (qRT-PCR). Total RNA was extracted and qRT-PCR was performed with a method modified as previously described. The primer sequences used for qRT-PCR are listed in Table S1. The relative gene expression level was calculated according to the 2^−ΔΔCt^ method. To visualize the relative expression levels data, YS was normalized as “1”. Three biological and three technical replicates were performed in these experiments.

### 2.6. Statistical Analysis

Statistical analysis of variance (ANOVA) with Duncan’s new multiple range test was carried out to compare cultivar mean values using SPSS Version 16.0 (Chicago, IL, USA). The significance level was set to *p* < 0.01. Graph Pad Prism version 6.0 (Graph Pad Software, San Diego, CA, USA) and Photoshop CS5 (Microsoft, Redmond, WA, USA and Adobe, San Jose, CA, USA) were used for graph plotting.

## 3. Results

### 3.1. Total Anthocyanin, Chlorophyll, Carotenoids, and Flavonoid Levels

**Figure 1:**
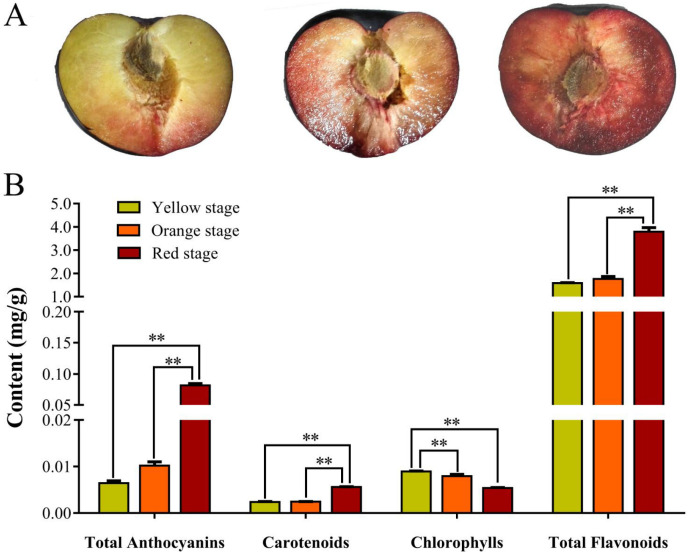
The phenotype of ‘Huaxiu’ plums fruits and pigment contents in the pulp. (**A**): Images of ‘Huaxiu’ plums fruits pulp at different coloration stages. (**B**): Contents of total anthocyanin, chlorophylls, carotenoids, and total flavonoids in the pulp of three coloration stages. The vertical bars represent the standard error of triplicate experiments. ** indicates significant differences in comparison to stems at *p* < 0.01.

As indicated in Figure 1A, the pulp color of ‘Huaxiu’ plums changed from yellow to red during ripening and these coincided with changes in the abundance of total anthocyanins, chlorophyll, carotenoids, and flavonoid in the pulp (Figure 1B). We determined that the total anthocyanins content of ‘Huaxiu’ plums increased from 6.45 × 10^−3^ mg/g FW at the pulp yellow stage (YS) to 81.52 × 10^−3^ mg/g FW at the pulp red stage (RS) as the ripening pulp color proceeded. The chlorophyll content was decreased from 8.94 × 10^−3^ mg/g at YS to 5.4 × 10^−3^ mg/g at RS, with a constant increase of the carotenoids content from 2.39 × 10^−3^ mg/g at YS to 5.61 × 10^−3^ mg/g at RS. The levels of flavonoids increased continuously during the change of the pulp color, and the contents were 1.59 mg/g at YS, 1.76 mg/g at OS, and 3.79 mg/g at RS, respectively.

### 3.2. Transcriptome Sequencing and De Novo Assembly

Three cDNA libraries were constructed from the total RNA of ‘Huaxiu’ plums pulp at yellow, orange, and red stage, we obtained 56,019,255, 58,211,832, and 55,985,990 raw reads, respectively, which consist of 8,380,734,292 bp, 8,713,283,611 bp, and 8,379,336,001 bp (Table S2). In addition, the Q20 of all pulps exceeded 97% and the GC content was around 46% (Table S2).

**Figure 2:**
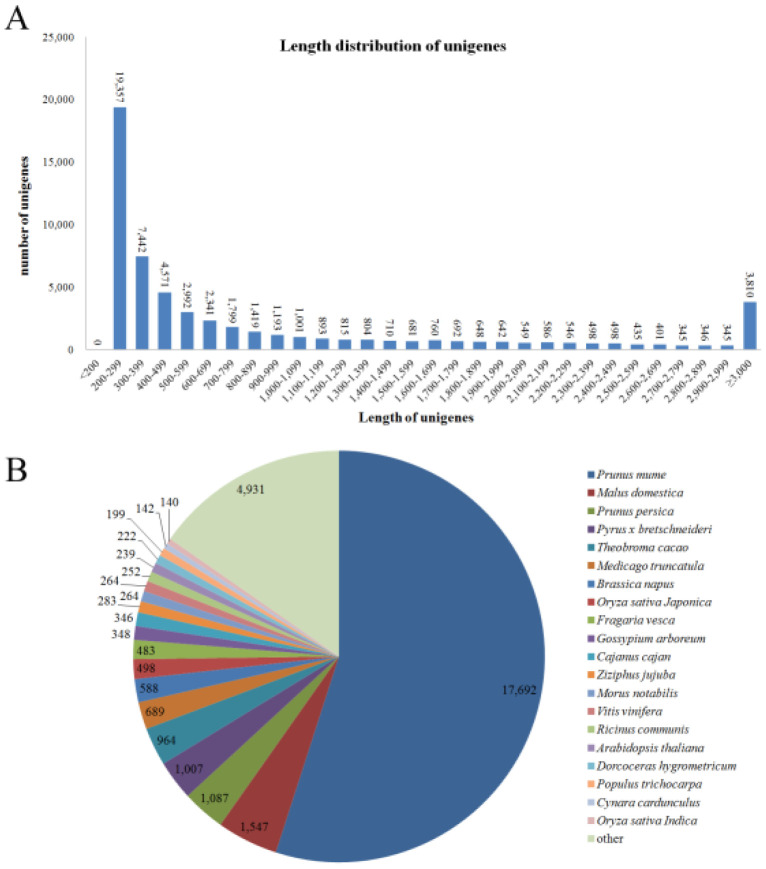
The distribution of the unigenes in ‘Huaxiu’ plums fruit transcriptome. (**A**): Sequence length distribution of the unigenes in ‘Huaxiu’ plums fruit transcriptome. The *x*-axis indicates unigene length interval from 200 bp to >3000 bp. The *y*-axis indicates the number of unigenes of each sequence length. (**B**): Results of species distribution of ‘Huaxiu’ plums unigenes top BLAST hits for all homologous sequences in NR (NCBI nonredundant) database. Different species are indicated by different colors.

After the removal of adaptor sequences, ambiguous reads, and low-quality reads for the assembly, a total of 57,119 unigenes consisting of 54,447,493 bp have been acquired. The percentage GC of all the unigenes was 41.07%, and the average length of unigene was 953 bp, the largest and smallest unigenes were 16,373 and 201 bp in length, respectively, and the N50 value was 1997 bp (Table 1). The results show that the sequences of unigenes are mostly concentrated between 200 and 399 bp and 400 and 799 bp, accounting for 46.9% and 20.5% of the total unigenes, respectively (Figure 2A and Table S3).

### 3.3. Functional Annotation of Unigenes

To annotate the transcriptome of ‘Huaxiu’ plums, 57,119 unigenes were searched against four public databases (NR, SwissProt, KOG, and KEGG) with a cutoff E-value of 10^−5^ (Figure S1 and Table S4). The functional annotation results showed only 61.6% of the unigenes (35,181) were identified (Table S4). Different unigenes have been matched in different databases, and 32,185 unique sequences were annotated with reference to the NR database, while 2996 unigenes were annotated using the other databases (Table S4).

The species distribution of ‘Huaxiu’ plum unigenes in the NR database is the best match, and showed the top matches with *Prunus mume* (55.0%), followed by *Malus domestica* (4.8%), *Prunus persica* (3.4%), *Pyrus bretschneideri* (3.1%), *Theobroma cacao* (3.0%), *Medicago truncatula* (2.1%), *Brassica napus* (1.8%), *Oryza sativa Japonica* (1.5%), *Fragaria vesca* (1.5%), *Gossypium arboreum* (1.1%), and *Cajanus cajan* (1.1%) (Figure 2B and Table S5).

### 3.4. Functional Classification of Unigenes

**Figure 3:**
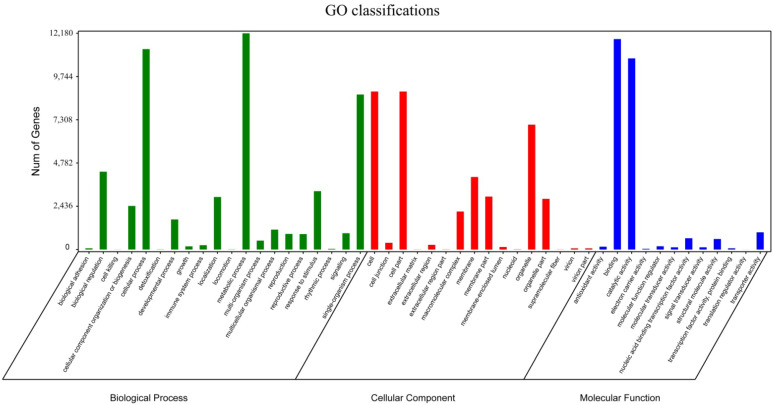
Gene ontology (GO) term level 2 categories of unigenes of the ‘Huaxiu’ plums (*P. salicina*).

Functional classification of unigenes by GO assignments resulted in the successful annotation of 21,438 unigenes. These unigenes were classified into three main GO categories, including biological process, molecular function, and cellular components (Figure 3, Table S6). Under the biological process category, metabolic process (12,176), cellular process (11,287), and single-organism process (8730) were the most dominant subcategories; the molecular function category mainly consisted of binding (11,852) and catalytic activity (10,764); the cellular components category mainly consisted of cell (8896), cell part (8895), organelle (7031), and membrane (4084).

**Figure 4:**
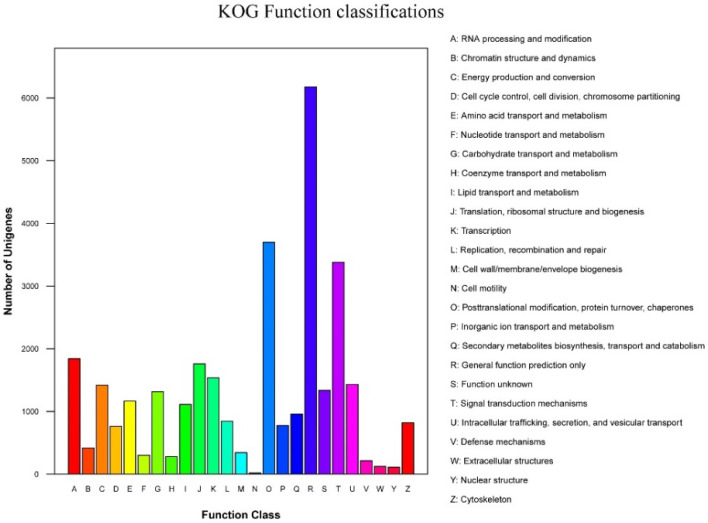
KOG functional classification of ‘Huaxiu’ plums (*P. salicina*) unigenes.

The validity of the plum transcriptome were further evaluated by KOG annotation, whereby 32,146 unigenes sequences were clustered into 25 KOG categories (Figure 4, Table S7). Among the 25 categories, which were alphabetized, the cluster of R (general function prediction only, 6176, 19.2%), group O (post-translational modification, protein turnover, and chaperones, 3697, 11.5%), and group T (signal transduction mechanisms, 3380, 10.5%) represented the three most abundant groups in the data. The group T (cell motility, 20, 0.1%) represented the smallest group.

To better understand biological pathways of ‘Huaxiu’ plum fruits’ pulp color change, all unigenes were searched in the KEGG database. A total of 7595 unigenes sequences were mapped onto 128 KEGG pathways in our results (Table S8). The pathways with the five highest unigenes representations were those related with the ribosome (ko03010; 644; 8.48%), carbon metabolism (ko01200; 629; 8.28%), biosynthesis of amino acids (ko01230; 517; 6.81%), protein processing in endoplasmic reticulum (ko04141; 387; 5.1%), and plant–pathogen interaction (ko04626; 322; 4.24%). Among these, carotenoids biosynthesis (ko00906; 45; 0.59%), flavonoid biosynthesis (ko00941; 44; 0.58%), and anthocyanin biosynthesis (ko00942; 1; 0.01%) appeared to be the smallest groups.

### 3.5. Differential Gene Expression between Three Stages of Pulp Coloration

**Figure 5:**
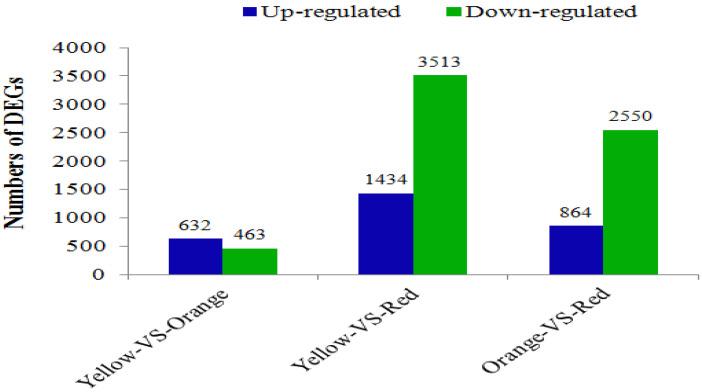
The numbers of DEGs in comparisons of the YS-vs.-OS, OS-vs.-RS, and YS-vs.-RS fruit samples.

To study unigene expression during different stages, the reads were mapped to the assembled transcriptome. A total of 56,570 unigenes (99%) were detected at all three stages. Among them, 50,987, 49,626, and 49,619 unigenes were detected in three pulp discoloration stages, respectively. In the results, 1095 transcripts were expressed differentially in yellow pulp compared to orange pulp, and among these genes, 632 were upregulated and 463 were downregulated (Figure 5 and Table S9). A total of 4947 shared DEGs were identified during the comparison between yellow and red, and among these DEGs, 1434 were unregulated and 3513 were down-regulated (Figure 5 and Table S11). Moreover, 3414 differentially expressed unigenes (864 unregulated and 2550 down regulated) were found between orange and red (Figure 5 and Table S10).

**Figure 6:**
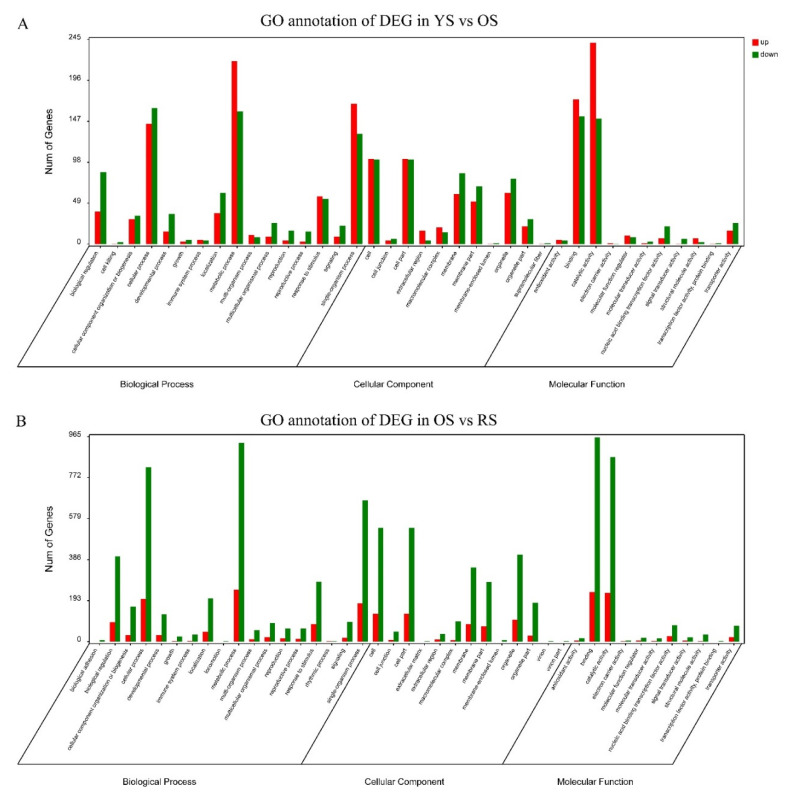

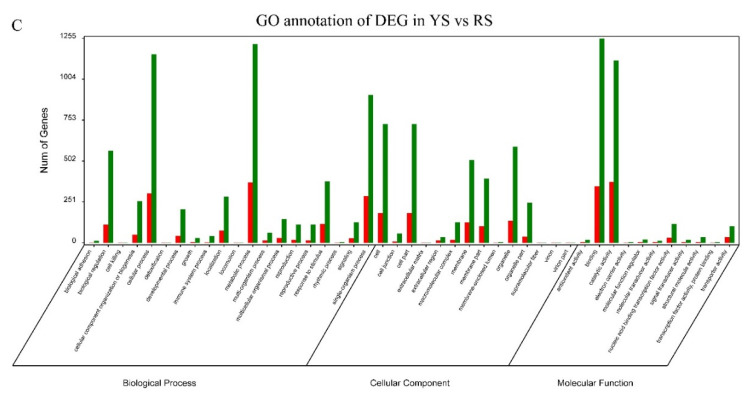
GO annotation of up- and downregulated expression genes of ‘Huaxiu’ plums (*P. salicina*) unigenes. (**A**): GO annotation in YS-vs.-OS; (**B**): GO annotation in OS-vs.-RS; (**C**): GO annotation in YS-vs.-RS.

The significant DEGs were represented in the three GO categories using the GO database (Figure 6). The results showed that five pathway (single-organism process, metabolic process, cellular process, catalytic activity, and binding) genes were most significantly enriched in the GO category. To annotate the functions of the unigene, we conducted a pathway-enrichment analysis of the DEGs in YS-vs.-OS, OS-vs.-RS, and YS-vs.-RS based on the KEGG database. According to the *p*-value statistics of the KEGG pathway enrichment, the top 20 pathways (*p*-values < 0.05) with the number of DEGs in the three periods are listed (Figure S2, Table S12). In YS-vs.-OS, the results showed that 218 DEGs were significantly enriched and were associated with 84 pathways, and among them, 10 pathways indicated the most significant enrichment, which mainly includes phenylpropanoid biosynthesis (18, 8.26%), starch and sucrose metabolism (21, 9.63%), plant hormone signal transduction (14, 6.42%), and plant–pathogen interaction (15, 6.88%) (Figure S2A, Table S12-1). In OS-vs.-RS, 580 DEGs were significantly enriched in 113 pathways, of which 19 pathways indicated the most significant enrichment. The following pathways are the most important in OS-vs.-RS: starch and sucrose metabolism (51, 8.79%), plant hormone signal transduction (39, 6.72%), plant–pathogen interaction (45, 7.76%), and phenylpropanoid biosynthesis (29, 5.00%) (Figure S2B, Table S12-2). In YS-vs.-RS, 750 DEGs were significantly enriched in 117 pathways, of which 22 pathways indicated the most significant enrichment. The following pathways are the most important in YS-vs.-RS: starch and sucrose metabolism (72, 9.6%), plant hormone signal transduction (55, 7.33%), plant–pathogen interaction (62, 8.27%), and pentose and glucuronate interconversions (29, 3.87%) (Figure S2C, Table S12-3).

### 3.6. Candidate Genes Related to Anthocyanin and Flavonoid Pathways

To identify unigenes involved in anthocyanin and flavonoid biosynthesis, KEGG functional enrichment was analyzed to characterize the functions of differentially expressed unigenes (Table 2). A total of 20 genes involved in anthocyanin and flavonoid biosynthesis were differentially expressed during ripening of ‘Huaxiu’ plums and only 1 DEG (Unigene0032408) was associated with the anthocyanin biosynthetic pathway, which was significantly upregulated during OS-vs.-RS. A total of 6, 12, and 17 DEGs were annotated as being involved in flavonoid biosynthesis in YS-vs.-OS, OS-vs.-RS, and YS-vs.-RS, respectively, with 3 DEGs common to the three sample groups. These structural genes of the flavonoid and anthocyanin biosynthetic pathways were significantly upregulated, including beta-D-glucosyl crocetin beta-1, 6-glucosyltransferase-like (Unigene0032408), CHS (Unigene0001957, Unigene0043265, Unigene0043266), CHI (Unigene0026465), DFR (Unigene0012798), LDOX (Unigene0001105), F3H (Unigene0002347, Unigene0040558), F3′H (Unigene0027142), and leucoanthocyanidin reductase-like (Unigene0025372), while other genes were downregulated. Structural genes CHS (Unigene0001957), CHS (Unigene0043265), DFR (Unigene0012798), and leucoanthocyanidin reductase-like (Unigene0025372) were the most significant DEGs in YS-vs-RS group, which showed 3.44-, 5.25-, 3.47-, and 5.33-fold upregulation, respectively. In addition, two genes (Unigene0002731 and Unigene0039938) were downregulated by 4.09- and 2.89-fold in YS-vs-RS, respectively.

To validate the RNA-Seq results, we selected 12 DEGs (flavonoid biosynthetic pathway genes) (Supplementary Figure S6) and analyzed their expression levels in YS, OS, and RS using RT-qPCR (Figure S3). The expression level trends of these genes were consistent with the changes in the abundance as detected in the RNA-Seq data.

## 4. Discussion

The color of the pulp cannot only be attractive in appearance, but also has higher nutritional value [1]. Therefore, it is very important to elucidate the genetic mechanism of pulp color regulation. It is generally believed the color of the pulp is determined by the comprehensive performance of chlorophyll, carotenoids, anthocyanins, and other pigment substances. Anthocyanin biosynthesis is fairly complex and is associated with flavonoids [46]. In our study, it was found that, during the coloring process of the pulp of ‘Huaxiu’ plums, the chlorophyll was gradually degraded and the synthesis of anthocyanin increased (Figure 1). When the comprehensive performance of anthocyanins and carotenoids made the pulp orange, then anthocyanins were synthesized in large amounts, the color of carotenoids was masked by anthocyanins, and the pulp was red. The main objective of this study was to identify the genes involved in anthocyanin biosynthesis in plums.

In this study, we have performed transcriptome sequencing of the stages of three fruit pulp colors of ‘Huaxiu’ plums with the use of advanced high-throughput Illumina RNA-Seq technology. The results not only enrich the gene information of *P. salicina*, but also can help explore the molecular genetics and biochemical characteristics of *Prunus salicina* Lindl. and its related species with the generated transcriptome data. In total, 57,119 unigenes were assembled, with a mean length of 953 bp (Table 1), which is comparable to 944 bp for sweet cherry (*P. avium* L.) [47], and with the longer to the previously reported other species such as 872 bp for cultivar ‘Furongli’ (*P. salicina* L.) [1] and 531 bp for Chinese bayberry (*Myrica rubra*) [48]. Approximately 61.6% of the unigenes were annotated to public databases (NR, Swiss-Prot, GO, COG, and KEGG), which means that more than one-third of the unigenes have no apparent homologs, with similar results seen in other no model plant species [49]. The unannotated unigenes could be plum-specific genes with novel functions, which may be related with some unique biosynthesis processes and pathways in the results. Furthermore, the annotated unigenes of *P. salicina* L. indicated the highest homology to those of *Prunus mume* (55.0%), followed by *Malus domestica* (4.8%) and *Prunus persica* (3.4%) (Figure 2B and Table S4), which may indicate the evolutionary relationship among these species. In spite of a large number of unigene sequences that indicated no matches, many of the unigenes were still assigned to a wide range of GO and KEGG classifications. The KEGG function annotation analysis showed that 7595 unigenes were involved in 128 biosynthesis process. The largest number of unigenes was associated with the ribosome, carbon metabolism, and biosynthesis of amino acids. However, the smallest number of unigenes was associated with anthocyanin biosynthesis, which have only one matching unigene. All of these data contribute to the study of the metabolic and biosynthesis mechanisms in *P. salicina*.

The RNA-Seq analysis revealed that the numbers of DEGs differed at the coloration stages, and we identified 1095, 3414, and 4947 DEGs between yellow and orange, orange and red, and yellow and red stages, respectively (Figure 5). More DEGs were detected at the yellow and red stage than at the yellow and orange stage, suggesting greater changes in the pulp color during the final ripening stage. Anthocyanin, the most important metabolite in flavonoid production, is an essential nutritional component in *P. salicina* fruits and their products. In our study, the flavonoid pathways were significantly enriched in the KEGG pathway. We identified many DEGs between different stages of the pulp color involved in anthocyanin and flavonoid biosynthesis, which mainly were structural genes, including CHS, CHI, DFR, F3H, F3′H, and LDOX, and were significantly upregulated during the pulp color of yellow vs. red color stages (Table 2). These observations agree well with those qRT-PCR results mentioned above. This is in accordance with findings for other fruits. To date, most of the structural genes in the anthocyanin biosynthetic pathway are upregulated during the fruit development of red/green skin color mutations of pear [50]. Similarly, coordinated expression changes of ANS, DFR1, F3H, F3′H, and UFGT have also been demonstrated in differently colored plum, sweet cherry, Chinese bayberries, and other plants [47,51].

CHS is considered to be a key enzyme in the anthocyanin biosynthesis of *Rosaceous* plants, which have diverse functions such as defense against pathogens and pigment biosynthesis. CHS proteins have been found responsible for the red coloration in crabapple cultivars [52], and the CHS protein of Japanese morning glory was also found to enhance both flavonoid production and flower pigmentation [53]. In the present study, we found that two CHSs (Unigene0001957 and Unigene0043265) were significantly upregulated at each stage, with the highest expression in the red stage. However, they do not correlate exactly with the increased concentration of anthocyanin content and total flavonoids during ‘Huaxiu’ plum coloration (Figure 1). This was probably due to the complicated composition of flavonoids. DFR and LDOX/ANS are late anthocyanin biosynthetic genes. F3H was one of three main enzymes in the primary phases of the flavonoid pathway. Transcript levels of F3H were greater in RS than YS, and the upregulation of F3H genes in RS indicates that it contributed to the accumulation of anthocyanin, which led to the red pigmentation in the plums. The expression of F3H also proved that it could make apples red [40]. In this study, these two structural genes (Unigene0012798 and Unigene0001105) showed the highest expression levels in the red stage, with the highest anthocyanin concentration (Figure 1, Table 2), which is consistent with findings for apple skin [54]. Thus, we believe that these genes may be play an important role in regulating anthocyanin biosynthesis in ‘Huaxiu’ plum fruit pulp.

The red hue of plant organs is caused due to anthocyanins, and the accumulation of these pigments is also regulated by transcription factors (TFs). In Rosaceae species, MYBs play a critical role as key transcription factors for all of the anthocyanin biosynthetic pathway genes or for the regulation of single key genes in fruit and flower color formation, particularly MYB10 genes, which are responsible for part of the natural variation in anthocyanin colors [10]. The bHLH proteins and NAC proteins have also been reported to be involved in anthocyanin synthesis [1]. The exact roles of these candidate transcription factor should be investigated in further studies.

## 5. Conclusions

The pulp of plums has been the focus of studies associated with fruit taste, quality, and nutrition. In the current study, we used RNA-Seq to analyze changes in the transcriptome with the pulp color of ‘Huaxiu’ plums. A total of 57,119 unigenes with a mean length of 953 bp were generated, and 61.6% of them were annotated to public databases. This study provides a large collection of DEG associated with plum fruit maturation processes. In addition, candidate genes involved in anthocyanin biosynthesis and flavonoid biosynthesis were identified in the transcriptome dataset. Further studies are needed to determine whether the identified candidate genes are related to anthocyanin biosynthesis in plums. This study has enriched the genetic dates of plums and provided an important platform for studying pulp-ripening processes in plums, especially anthocyanin and flavonoid biosynthesis, which will lay the foundation for further functional genomics and molecular metabolic mechanism studies on plums.

## Figures and Tables

**Table 1:** Summary of sequencing and de novo assembly results of unigenes.

Type	Unigene
Genes Num	57,119
Total assembled bases	54,447,493
Percent GC (%)	41.07
Max length (bp)	16,373
Min length (bp)	201
Average length (bp)	953
N50	1997

**Table 2:** Pathways associated with candidate differentially expressed genes involved in anthocyanin biosynthesis and flavonoid biosynthesis in ‘Huaxiu’ plums.

Pathway	DEGs	KO ID	Description	log_2_ (OS/YS)	*p*-Value	log_2_ (RS/OS)	*p*-Value	log_2_ (RS/YS)	*p*-Value
**Anthocyanin biosynthesis**	Unigene0032408	ko00942	Beta-D-glucosyl crocetin beta-1,6-glucosyltransferase-like	—	—	1.31	1.01 × 10^−24^	—	—
**Flavonoid biosynthesis**	Unigene0001957	ko00941	Chalcone synthase (CHS)	1.79	3.19 × 10^−43^	1.65	1.83 × 10^−64^	3.44	5.17 × 10^−144^
	Unigene0043265	ko00941	Chalcone synthase (CHS)	3.33	1.04 × 10^−33^	1.92	2.01 × 10^−34^	5.25	1.47 × 10^−172^
	Unigene0043266	ko00941	Chalcone synthase (CHS)	—	—	1.90	0.0006	1.79	0.0004
	Unigene0026465	ko00941	Chalcone isomerase (CHI)	—	—	1.69	0.0006	1.46	2.26 × 10^−43^
	Unigene0012798	ko00941	Dihydroflavonol 4-reductase (DFR)	1.49	4.61 × 10^−7^	1.98	6.68 × 10^−20^	3.47	1.09 × 10^−55^
	Unigene0001105	ko00941	Leucoanthocyanidin dioxygenase (LDOX)	1.32	4.43 × 10^−27^	—	—	1.89	1.90 × 10^−61^
	Unigene0002347	ko00941	Flavonol synthase/flavanone 3-hydroxylase-like (FS/F3′H)	—	—	1.70	5.48 × 10^−14^	2.37	1.86 × 10^−32^
	Unigene0040558	ko00941	Flavanone 3-hydroxylase (F3H)	—	—	—	—	1.54	1.16 × 10^−56^
	Unigene0027142	ko00941	Flavonoid 3′-hydroxylase(F3′H)	—	—	1.19	1.22 × 10^−38^	1.00	5.62 × 10^−30^
	Unigene0022847	ko00940	Flavonoid 3′,5′-methyltransferase-like (F3′5′H)	—	—	—	—	−1.64	0.0035
	Unigene0023053	ko00940	Flavonoid 3′,5′-methyltransferase-like(F3′5′H)	—	—	—	—	−1.58	2.62 × 10^−7^
	Unigene0025278	ko00941	Leucoanthocyanidin reductase-like	—	—	−1.13	3.50 × 10^−5^	−1.03	1.09 × 10^−5^
	Unigene0025372	ko00941	Leucoanthocyanidin reductase-like isoform X1	—	—	3.82	6.66 × 10^−7^	5.33	3.46 × 10^−11^
	Unigene0039938	ko00941	Shikimate O-hydroxycinnamoyltransferase-like	—	—	—	—	−2.89	0.0059
	Unigene0039940	ko00941	Shikimate O-hydroxycinnamoyltransferase-like	−1.07	5.02 × 10^−15^	—	—	−1.45	7.34 × 10^−31^
	Unigene0039941	ko00941	Shikimate O-hydroxycinnamoyltransferase-like	—	—	−1.25	0.0005	−1.91	9.24 × 10^−11^
	Unigene0002731	ko00941	Shikimate O-hydroxycinnamoyltransferase-like	—	—	−3.75	0.0054	−4.09	0.0012
	Unigene0037918	ko00941	Acylsugar acyltransferase 3-like	1.26	0.0001	—	—	—	—
	Unigene0019559	ko00941	3,5-dihydroxybiphenyl synthase-like	—	—	−1.09	1.99 × 10^−7^	—	—

Note: —: The gene expression level that had no significant differences.

## Data Availability

All relevant data are within the paper and its Supplementary Materials.

## Supplementary Materials

The following supporting information can be downloaded at: https://www.mdpi.com/article/10.3390/cimb44120434/s1, Figure S1: The Venn diagram of unigene numbers annotated by BLASTx against four public databases; Figure S2: Pathway enrichment (top 20) of differentially expressed genes between the two colors stages of pulp; Figure S3: Expression analysis of DEG by qRT-PCR; Table S1: Primer sequences of unigenes used for quantitative RT-PCR verification; Table S2: Summary of read numbers based on the RNA-Seq data from the pulp of ‘Huaxiu’ pump during the yellow, orange and red stages of maturation; Table S3: The length distribution of the assembled unigenes; Table S4: Summary of functional annotations for the assembled unigenes; Table S5: The species distribution of unigene BLAST results against the nr database; Table S6: Summary of level2 GO term classification for the ‘Huaxiu’ plums (*P. salicina*) transcriptome; Table S7: Summary of KOG functional classification for the ‘Huaxiu’ plums (*P. salicina*) unigenes; Table S8: Summary of KEGG pathways enrichment involved in the ‘Huaxiu’ plums (*P. salicina*) unigenes; Table S9: Summary of significance DEGs in YS-vs.-OS; Table S10: Summary of significance DEGs in OS-vs.-RS; Table S11: Summary of significance DEGs in YS-vs.-RS; Table S12: Summary of pathway enrichment of DEGs between the two colors stages of pulp (top 20).

## Abbreviations

ANS	anthocyanidin synthase
bHLH	basic helix–loop–helix
CHS	chalcone synthase
COG	cluster of orthologous groups of proteins
CH	chalcone isomerase
DFR	dihydroflavonol 4-reductase
DGE	differentially expressed genes
F3H	flavanone 3-hydroxylase
F3’H	flavonoid 3’-hydroxylase
FDR	false discovery rate
FPKM	fragments per kilobase of transcript per million mapped reads
GO	gene ontology
KEGG	Kyoto Encyclopedia of Genes and Genomes
LDOX	leucoanthocyanidin dioxygenase
NR	NCBI nonredundant protein database
OS	orange stage
qRT-PCR	quantitative real-time PCR
RNA-Seq	RNA sequencing
RS	red stage
RSEM	RNA-Seq by expectation maximization
UFGT	UDP-glucose: flavonoid 3-O-glucosyltransferase
YS	yellow stage
