# Supplementary material for: Identification of Candidate Genes Associated with Pulp Color by Transcriptomic Analysis of ‘Huaxiu’ Plum (Prunus salicina Lindl.) during Fruit-Ripening
Source: Curr Issues Mol Biol. 2022 Dec 15;44(12):6368–84. doi: 10.3390/cimb44120434 (PMC9776821; doi:10.3390/cimb44120434)
Supplement: Supplementary file 1 [file cimb-44-00434-s001.zip › Table S1.pdf]

Table S1. Primer sequences of unigenes used for quantitative RT-PCR verification

Unigene name	Forward primer	Reverse primer
Unigene0001957 (CHS)	AGCAGCCCAAACCATCCTTCC	GCAATCCAGAATAGTGAGTTCCAATCC
Unigene0043265 (CHS)	ACATTGAATTTGAGGTGGGTTAGGTC	CCAGTGGCAAGGCAATACGC
Unigene0026465 (CHI)	AACAAATACATCTCCATCGCCACAC	TCCTCCAAGTACACGCCAATCG
Unigene0012798 (DFR)	AAATTGAACAAGCAAGCAGTCCATAC	CTCGTCCGCCAGGTCAGC
Unigene0001105 (LDOX)	GTTGTGGAGTATGAAGGTGAGTGC	TTGGGTTGGGATTGGAAGAAGGG
Unigene0002347 (F3'H)	AAAGTGATGGAGGTGGAGAGAGTG	CCGTGGTCAGTAATGAGAAAGAAGC
Unigene0040558 (F3H)	GAGCATCAGAGAGGGAGAGAAGC	CCAGTAGCATAACACGAGCAAGC
Unigene0027142 (F3'H)	AGGAGATGGTTGTGGAGATGATGG	GCCGCTGCTCTTCTTGTGTTC
Unigene0022847 (F3'5'H)	GTTGATGAAGGGCTGTTGCTCTC	AAGGCATCCGACTGAAAGAAGTTAATC
Unigene0025372	GCCGATGTTCTCCCACCCTTG	GCCGATGTTCTCCCACCCTTG
Unigene0039941	GTTCCCGCCCTATCACATACCC	AAGAGCACTCCCTCCGCATTG
Unigene0002731	GGCGGAGAAGGTGACAGTGAG	CTTGCTGAGGGCTTGCTTGAG
Actin	AACTGGGATGACATGGAGAAGATC	AATGGCATGAGGTAGGGCATAA
