# Supplementary material for: Identification of Candidate Genes Associated with Pulp Color by Transcriptomic Analysis of ‘Huaxiu’ Plum (Prunus salicina Lindl.) during Fruit-Ripening
Source: Curr Issues Mol Biol. 2022 Dec 15;44(12):6368–84. doi: 10.3390/cimb44120434 (PMC9776821; doi:10.3390/cimb44120434)
Supplement: Supplementary file 1 [file cimb-44-00434-s001.zip › Table S12.pdf]

Table S12-1. Pathway Enrichment in YS-vs-OS (top 20).

	KEGG Pathway	Up genes of DEGs (137)	Down genes of DEGs (81)	All DEGs genes with pathway (218)	Percentage	P-value
1	Phenylpropanoid biosynthesis	12	6	18	8.26%	0.000004
2	Starch and sucrose metabolism	16	5	21	9.63%	0.000074
3	Pentose and glucuronate interconversions	8	4	12	5.50%	0.000135
4	Diterpenoid biosynthesis	1	4	5	2.29%	0.001066
5	Flavonoid biosynthesis	5	1	6	2.75%	0.001480
6	Amino sugar and nucleotide sugar metabolism	9	3	12	5.50%	0.007148
7	Plant hormone signal transduction	6	8	14	6.42%	0.011492
8	Glutathione metabolism	9	-	9	4.13%	0.012867
9	Ether lipid metabolism	3	-	3	1.38%	0.041111
10	Plant-pathogen interaction	7	8	15	6.88%	0.043659
11	Fatty acid elongation	2	1	3	1.38%	0.053629
12	Isoquinoline alkaloid biosynthesis	2	1	3	1.38%	0.062872
13	Brassinosteroid biosynthesis	3	-	3	1.38%	0.083378
14	Cutin, suberine and wax biosynthesis	2	-	2	0.92%	0.111183
15	Endocytosis	5	5	10	4.59%	0.135490
16	Sesquiterpenoid and triterpenoid biosynthesis	2	-	2	0.92%	0.150145
17	Phenylalanine metabolism	3	1	4	1.83%	0.157101
18	Linoleic acid metabolism	1	1	2	0.92%	0.160271
19	Monoterpenoid biosynthesis	1	1	2	0.92%	0.180870
20	Stilbenoid, diarylheptanoid and gingerol biosynthesis	1	1	2	0.92%	0.191314

Table S12-2. Pathway Enrichment in OS-vs-RS (top 20).

	KEGG Pathway	Up genes of DEGs (138)	Down genes of DEGs (442)	All DEGs genes with pathway (580)	Percentage	P-value
1	Starch and sucrose metabolism	12	39	51	8.79%	0.000000
2	Plant hormone signal transduction	10	29	39	6.72%	0.000009
3	Plant-pathogen interaction	5	40	45	7.76%	0.000046
4	Flavonoid biosynthesis	8	4	12	2.07%	0.000076
5	Phenylpropanoid biosynthesis	6	23	29	5.00%	0.000098
6	Fatty acid elongation	6	2	8	1.38%	0.001427
7	Taurine and hypotaurine metabolism	4	2	6	1.03%	0.001619
8	Isoquinoline alkaloid biosynthesis		8	8	1.38%	0.002239
9	Cyanoamino acid metabolism	1	12	13	2.24%	0.002810
10	Ether lipid metabolism	1	6	7	1.21%	0.003363
11	Pentose and glucuronate interconversions	8	10	18	3.10%	0.003465
12	Diterpenoid biosynthesis	5	2	7	1.21%	0.004195
13	Tropane, piperidine and pyridine alkaloid biosynthesis	-	7	7	1.21%	0.009140
14	Glutathione metabolism	3	15	18	3.10%	0.010384
15	Zeatin biosynthesis	1	2	3	0.52%	0.018553
16	Galactose metabolism	2	11	13	2.24%	0.025711
17	Amino sugar and nucleotide sugar metabolism	3	19	22	3.79%	0.025903
18	alpha-Linolenic acid metabolism	6	5	11	1.90%	0.033814
19	Linoleic acid metabolism	1	4	5	0.86%	0.037799
20	Brassinosteroid biosynthesis	1	5	6	1.03%	0.052866

Table S12-3. Pathway Enrichment in YS-vs-RS (top 20).

	KEGG Pathway	Up genes of DEGs (208)	Down genes of DEGs (542)	All DEGs genes with pathway (750)	Percentage	P-value
1	Starch and sucrose metabolism	20	52	72	9.6%	0.000000
2	Plant hormone signal transduction	16	39	55	7.3%	0.000000
3	Plant-pathogen interaction	8	54	62	8.3%	0.000000
4	Flavonoid biosynthesis	10	7	17	2.3%	0.000000
5	Pentose and glucuronate interconversions	13	16	29	3.9%	0.000003
6	Phenylpropanoid biosynthesis	12	25	37	4.9%	0.000012
7	Taurine and hypotaurine metabolism	5	3	8	1.1%	0.000155
8	Galactose metabolism	2	17	19	2.5%	0.001801
9	Fatty acid elongation	7	2	9	1.2%	0.001806
10	Glutathione metabolism	8	14	22	2.9%	0.008852
11	Isoquinoline alkaloid biosynthesis	-	8	8	1.1%	0.010704
12	Ether lipid metabolism	1	6	7	0.9%	0.013582
13	Stilbenoid, diarylheptanoid and gingerol biosynthesis	-	7	7	0.9%	0.016608
14	alpha-Linolenic acid metabolism	7	7	14	1.9%	0.019296
15	Cyanoamino acid metabolism	2	11	13	1.7%	0.022590
16	Phenylalanine metabolism	3	10	13	1.7%	0.025095
17	Photosynthesis	1	20	21	2.8%	0.025263
18	Tyrosine metabolism	2	11	13	1.7%	0.030711
19	Linoleic acid metabolism	2	4	6	0.8%	0.031397
20	Tropane, piperidine and pyridine alkaloid biosynthesis	-	7	7	0.9%	0.033461

Note: -.The pathway was not significant
