# Supplementary material for: Identification of Candidate Genes Associated with Pulp Color by Transcriptomic Analysis of ‘Huaxiu’ Plum (Prunus salicina Lindl.) during Fruit-Ripening
Source: Curr Issues Mol Biol. 2022 Dec 15;44(12):6368–84. doi: 10.3390/cimb44120434 (PMC9776821; doi:10.3390/cimb44120434)
Supplement: Supplementary file 1 [file cimb-44-00434-s001.zip › Table S2.pdf]

Table S2. Summary of read numbers based on the RNA-Seq data from the pulp of 'Huaxiu' pump during the yellow, orange and red stages of maturation

Summary	Yellow			Orange			Red		
	YS-1	YS-2	Mean	OS-1	OS-2	Mean	RS-1	RS-2	Mean
All Reads Num	55840988	56197522	56019255	59505786	56917878	58211832	59849866	52122114	55985990
GC Percentage	45.92%	45.81%	45.87%	45.68%	45.8%	45.74%	46%	45.98%	45.99%
Bases after Filter	8351322365	8410146219	8380734292	8907146341	8519420880	8713283611	8956546472	7802125530	8379336001
Q20 Percentage	97.26%	97.06%	97.16%	97.03%	97.23%	97.13%	96.97%	97.34%	97.16%
Q30 Percentage	92.94%	92.50%	92.72%	92.41%	92.85%	92.63%	92.28%	93.07%	92.68%
Total mapped reads	49002007	49331284	49536759	52309871	49665744	514078879	52831026	45992106	49679550
Total mapped reads Ratio	88.45 %	88.41 %	88.43%	88.42 %	88.20 %	88.31%	88.79 %	88.67 %	88.74%
Unique Mapped Reads	47141180	47503820	47322500	50106969	47586762	48846865.5	50375983	43822669	47099326
Unique Mapped Reads Ratio	85.09%	85.13%	85.11%	84.70%	84.51%	84.60%	84.67%	84.49%	84.58%
Multiple Mapped Reads	1860827	1827464	1844146	2202902	2078982	2140942	2455043	2169437	2312240
Multiple Mapped Reads Ratio	3.36%	3.27%	3.32%	3.72%	3.69%	3.71%	4.13%	4.18%	4.15%
Unmapped Reads	6398959	6469166	6434063	6850255	6646438	6748347	6667962	5876880	6272421
Unmapped Reads Ratio	11.55%	11.59%	11.57%	11.58%	11.80%	11.69%	11.21%	11.33%	11.26%

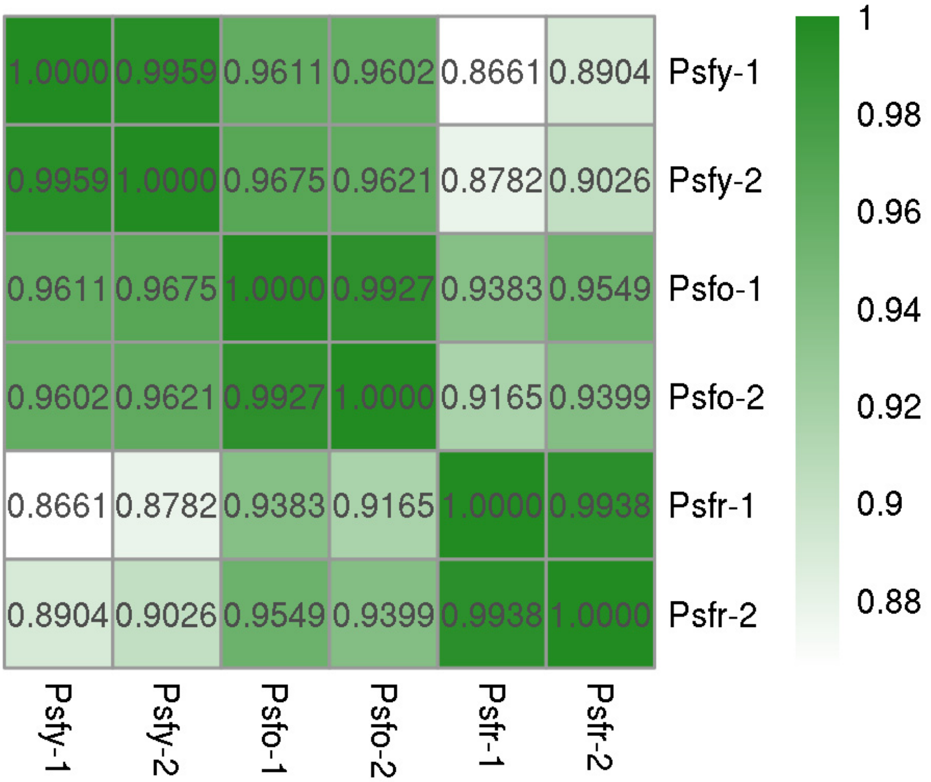

The correlation analysis of RNA-Seq data of 'Huaxiu' pump during the yellow, orange and red stages.

Psfy: yellow stage (YS); Psfo: orange stage (OS); Psfr: Red stage (RS).
