# Supplementary material for: Identification of Candidate Genes Associated with Pulp Color by Transcriptomic Analysis of ‘Huaxiu’ Plum (Prunus salicina Lindl.) during Fruit-Ripening
Source: Curr Issues Mol Biol. 2022 Dec 15;44(12):6368–84. doi: 10.3390/cimb44120434 (PMC9776821; doi:10.3390/cimb44120434)
Supplement: Supplementary file 1 [file cimb-44-00434-s001.zip › Table S3.pdf]

Table S3. The length distribution of the assembled unigenes

Length of unigene	Number of unigenes	Percentage	Total percentage
<200	0	0.0%	0.0%
200-299	19357	33.9%	46.9%
300-399	7442	13.0%	
400-499	4571	8.0%	20.5%
500-599	2992	5.2%	
600-699	2341	4.1%	
700-799	1799	3.1%	
800-899	1419	2.5%	18.0%
900-999	1193	2.1%	
1000-1099	1001	1.8%	
1100-1199	893	1.6%	
1200-1299	815	1.4%	
1300-1399	804	1.4%	
1400-1499	710	1.2%	
1500-1599	681	1.2%	
1600-1699	760	1.3%	
1700-1799	692	1.2%	
1800-1899	648	1.1%	
1900-1999	642	1.1%	
2000-2099	549	1.0%	8.0%
2100-2199	586	1.0%	
2200-2299	546	1.0%	
2300-2399	498	0.9%	
2400-2499	498	0.9%	
2500-2599	435	0.8%	
2600-2699	401	0.7%	
2700-2799	345	0.6%	

2800-2899	346	0.6%	
2900-2999	345	0.6%	
⩾3000	3810	6.7%	6.7%
