# Supplementary material for: Identification of Candidate Genes Associated with Pulp Color by Transcriptomic Analysis of ‘Huaxiu’ Plum (Prunus salicina Lindl.) during Fruit-Ripening
Source: Curr Issues Mol Biol. 2022 Dec 15;44(12):6368–84. doi: 10.3390/cimb44120434 (PMC9776821; doi:10.3390/cimb44120434)
Supplement: Supplementary file 1 [file cimb-44-00434-s001.zip › Table S4.pdf]

Table S4. Summary of functional annotations for the assembled unigenes

Database	Number of annotated unigenes	Percentage of annotated unigenes (%)
Total Unigenes	57119	
Nr	32185	56.3%
Swissprot	25299	44.3%
KOG	19121	33.5%
KEGG	13143	23.0%
Annotation genes	35181	61.6%
