# Supplementary material for: Identification of Candidate Genes Associated with Pulp Color by Transcriptomic Analysis of ‘Huaxiu’ Plum (Prunus salicina Lindl.) during Fruit-Ripening
Source: Curr Issues Mol Biol. 2022 Dec 15;44(12):6368–84. doi: 10.3390/cimb44120434 (PMC9776821; doi:10.3390/cimb44120434)
Supplement: Supplementary file 1 [file cimb-44-00434-s001.zip › Table S5 .pdf]

**Table S5.** The species distribution of unigene BLAST results against the nr database

| Species | Unigene Num | Percentage |
|---|---|---|
| *Prunus mume* | 17692 | 55.0% |
| *Malus domestica* | 1547 | 4.8% |
| *Prunus persica* | 1087 | 3.4% |
| *Pyrus x bretschneideri* | 1007 | 3.1% |
| *Theobroma cacao* | 964 | 3.0% |
| *Medicago truncatula* | 689 | 2.1% |
| *Brassica napus* | 588 | 1.8% |
| *Oryza sativa Japonica* | 498 | 1.5% |
| *Fragaria vesca* | 483 | 1.5% |
| *Gossypium arboreum* | 348 | 1.1% |
| *Cajanus cajan* | 346 | 1.1% |
| *Ziziphus jujuba* | 283 | 0.9% |
| *Morus notabilis* | 264 | 0.8% |
| *Vitis vinifera* | 264 | 0.8% |
| *Ricinus communis* | 252 | 0.8% |
| *Arabidopsis thaliana* | 239 | 0.7% |
| *Dorcoceras hygrometricum* | 222 | 0.7% |
| *Populus trichocarpa* | 199 | 0.6% |
| *Cynara cardunculus* | 142 | 0.4% |
| *Oryza sativa Indica* | 140 | 0.4% |
| other | 4931 | 15.3% |
