# Supplementary material for: Identification of Candidate Genes Associated with Pulp Color by Transcriptomic Analysis of ‘Huaxiu’ Plum (Prunus salicina Lindl.) during Fruit-Ripening
Source: Curr Issues Mol Biol. 2022 Dec 15;44(12):6368–84. doi: 10.3390/cimb44120434 (PMC9776821; doi:10.3390/cimb44120434)
Supplement: Supplementary file 1 [file cimb-44-00434-s001.zip › Table S8.pdf]

Table S8. Summary of KEGG pathways enrichment involved in the 'Huaxiu' plums (*P. salicina*) unigenes

	Pathway	All genes with pathway annotation (7595)	Pathway ID
1	Ribosome	644 (8.48%)	ko03010
2	Carbon metabolism	629 (8.28%)	ko01200
3	Biosynthesis of amino acids	517 (6.81%)	ko01230
4	Protein processing in endoplasmic reticulum	387 (5.1%)	ko04141
5	Plant-pathogen interaction	322 (4.24%)	ko04626
6	Spliceosome	322 (4.24%)	ko03040
7	Oxidative phosphorylation	308 (4.06%)	ko00190
8	RNA transport	299 (3.94%)	ko03013
9	Starch and sucrose metabolism	288 (3.79%)	ko00500
10	Glycolysis / Gluconeogenesis	275 (3.62%)	ko00010
11	Plant hormone signal transduction	247 (3.25%)	ko04075
12	Purine metabolism	238 (3.13%)	ko00230
13	Endocytosis	234 (3.08%)	ko04144
14	Carbon fixation in photosynthetic organisms	229 (3.02%)	ko00710
15	Ubiquitin mediated proteolysis	211 (2.78%)	ko04120
16	Glyoxylate and dicarboxylate metabolism	200 (2.63%)	ko00630
17	Amino sugar and nucleotide sugar metabolism	186 (2.45%)	ko00520
18	Pyrimidine metabolism	185 (2.44%)	ko00240
19	Pyruvate metabolism	185 (2.44%)	ko00620
20	Phenylpropanoid biosynthesis	181 (2.38%)	ko00940
21	Peroxisome	180 (2.37%)	ko04146
22	mRNA surveillance pathway	174 (2.29%)	ko03015
23	RNA degradation	170 (2.24%)	ko03018
24	Cysteine and methionine metabolism	169 (2.23%)	ko00270
25	Phagosome	164 (2.16%)	ko04145
26	Glycine, serine and threonine metabolism	154 (2.03%)	ko00260
27	Photosynthesis	136 (1.79%)	ko00195
28	Ribosome biogenesis in eukaryotes	133 (1.75%)	ko03008
29	Fatty acid metabolism	132 (1.74%)	ko01212
30	Glutathione metabolism	131 (1.72%)	ko00480
31	Aminoacyl-tRNA biosynthesis	125 (1.65%)	ko00970
32	Glycerophospholipid metabolism	123 (1.62%)	ko00564
33	Citrate cycle (TCA cycle)	122 (1.61%)	ko00020
34	Pentose phosphate pathway	118 (1.55%)	ko00030
35	Pentose and glucuronate interconversions	118 (1.55%)	ko00040
36	Alanine, aspartate and glutamate metabolism	117 (1.54%)	ko00250
37	Proteasome	117 (1.54%)	ko03050
38	2-Oxocarboxylic acid metabolism	110 (1.45%)	ko01210
39	Fructose and mannose metabolism	107 (1.41%)	ko00051

40	Valine, leucine and isoleucine degradation	102 (1.34%)	ko00280
41	Phosphatidylinositol signaling system	97 (1.28%)	ko04070
42	Fatty acid degradation	97 (1.28%)	ko00071
43	Glycerolipid metabolism	96 (1.26%)	ko00561
44	Arginine and proline metabolism	95 (1.25%)	ko00330
45	Galactose metabolism	94 (1.24%)	ko00052
46	Nucleotide excision repair	94 (1.24%)	ko03420
47	Protein export	90 (1.18%)	ko03060
48	Propanoate metabolism	87 (1.15%)	ko00640
49	Inositol phosphate metabolism	87 (1.15%)	ko00562
50	beta-Alanine metabolism	87 (1.15%)	ko00410
51	Homologous recombination	85 (1.12%)	ko03440
52	Ascorbate and aldarate metabolism	83 (1.09%)	ko00053
53	Porphyrin and chlorophyll metabolism	83 (1.09%)	ko00860
54	Photosynthesis - antenna proteins	82 (1.08%)	ko00196
55	DNA replication	81 (1.07%)	ko03030
56	Arginine biosynthesis	80 (1.05%)	ko00220
57	Terpenoid backbone biosynthesis	79 (1.04%)	ko00900
58	Fatty acid biosynthesis	79 (1.04%)	ko00061
59	alpha-Linolenic acid metabolism	78 (1.03%)	ko00592
60	Tyrosine metabolism	75 (0.99%)	ko00350
61	Phenylalanine metabolism	73 (0.96%)	ko00360
62	Cyanoamino acid metabolism	72 (0.95%)	ko00460
63	Phenylalanine, tyrosine and tryptophan biosynthesis	72 (0.95%)	ko00400
64	Nitrogen metabolism	70 (0.92%)	ko00910
65	Base excision repair	69 (0.91%)	ko03410
66	Ubiquinone and other terpenoid-quinone biosynthesis	67 (0.88%)	ko00130
67	ABC transporters	67 (0.88%)	ko02010
68	Lysine degradation	66 (0.87%)	ko00310
69	N-Glycan biosynthesis	66 (0.87%)	ko00510
70	Mismatch repair	65 (0.86%)	ko03430
71	RNA polymerase	64 (0.84%)	ko03020
72	Basal transcription factors	63 (0.83%)	ko03022
73	Sulfur metabolism	62 (0.82%)	ko00920
74	Circadian rhythm - plant	59 (0.78%)	ko04712
75	Tryptophan metabolism	59 (0.78%)	ko00380
76	Sphingolipid metabolism	56 (0.74%)	ko00600
77	Biosynthesis of unsaturated fatty acids	54 (0.71%)	ko01040
78	Regulation of autophagy	52 (0.68%)	ko04140
79	Butanoate metabolism	52 (0.68%)	ko00650
80	Pantothenate and CoA biosynthesis	52 (0.68%)	ko00770
81	SNARE interactions in vesicular transport	49 (0.65%)	ko04130

82	Selenocompound metabolism	49 (0.65%)	ko00450
83	Histidine metabolism	47 (0.62%)	ko00340
84	Carotenoid biosynthesis	45 (0.59%)	ko00906
85	Flavonoid biosynthesis	44 (0.58%)	ko00941
86	Glycosylphosphatidylinositol(GPI)-anchor biosynthesis	43 (0.57%)	ko00563
87	Valine, leucine and isoleucine biosynthesis	42 (0.55%)	ko00290
88	One carbon pool by folate	39 (0.51%)	ko00670
89	Brassinosteroid biosynthesis	36 (0.47%)	ko00905
90	Nicotinate and nicotinamide metabolism	36 (0.47%)	ko00760
91	Other glycan degradation	36 (0.47%)	ko00511
92	Steroid biosynthesis	36 (0.47%)	ko00100
93	Biotin metabolism	35 (0.46%)	ko00780
94	Lysine biosynthesis	33 (0.43%)	ko00300
95	Tropane, piperidine and pyridine alkaloid biosynthesis	32 (0.42%)	ko00960
96	Isoquinoline alkaloid biosynthesis	32 (0.42%)	ko00950
97	Glycosaminoglycan degradation	30 (0.39%)	ko00531
98	Fatty acid elongation	30 (0.39%)	ko00062
99	Diterpenoid biosynthesis	28 (0.37%)	ko00904
100	Stilbenoid, diarylheptanoid and gingerol biosynthesis	28 (0.37%)	ko00945
101	Ether lipid metabolism	27 (0.36%)	ko00565
102	Monoterpenoid biosynthesis	27 (0.36%)	ko00902
103	Arachidonic acid metabolism	25 (0.33%)	ko00590
104	Linoleic acid metabolism	25 (0.33%)	ko00591
105	Sesquiterpenoid and triterpenoid biosynthesis	24 (0.32%)	ko00909
106	Folate biosynthesis	24 (0.32%)	ko00790
107	Sulfur relay system	23 (0.3%)	ko04122
108	Thiamine metabolism	21 (0.28%)	ko00730
109	C5-Branched dibasic acid metabolism	20 (0.26%)	ko00660
110	Cutin, suberine and wax biosynthesis	20 (0.26%)	ko00073
111	Vitamin B6 metabolism	19 (0.25%)	ko00750
112	Monobactam biosynthesis	19 (0.25%)	ko00261
113	Taurine and hypotaurine metabolism	18 (0.24%)	ko00430
114	Degradation of aromatic compounds	17 (0.22%)	ko01220
115	Riboflavin metabolism	16 (0.21%)	ko00740
116	Glycosphingolipid biosynthesis - globo series	15 (0.2%)	ko00603
117	Non-homologous end-joining	14 (0.18%)	ko03450
118	Synthesis and degradation of ketone bodies	11 (0.14%)	ko00072
119	Glycosphingolipid biosynthesis - ganglio series	9 (0.12%)	ko00604
120	Zeatin biosynthesis	8 (0.11%)	ko00908
121	Lipoic acid metabolism	8 (0.11%)	ko00785
122	Caffeine metabolism	4 (0.05%)	ko00232

123	Flavone and flavonol biosynthesis	4 (0.05%)	ko00944
124	Other types of O-glycan biosynthesis	4 (0.05%)	ko00514
125	Glucosinolate biosynthesis	3 (0.04%)	ko00966
126	Limonene and pinene degradation	3 (0.04%)	ko00903
127	Anthocyanin biosynthesis	1 (0.01%)	ko00942
128	Isoflavonoid biosynthesis	1 (0.01%)	ko00943
