# Supplementary figures and images for: Identification of Candidate Genes Associated with Pulp Color by Transcriptomic Analysis of ‘Huaxiu’ Plum (Prunus salicina Lindl.) during Fruit-Ripening
Source: Curr Issues Mol Biol. 2022 Dec 15;44(12):6368–84. doi: 10.3390/cimb44120434 (PMC9776821; doi:10.3390/cimb44120434)

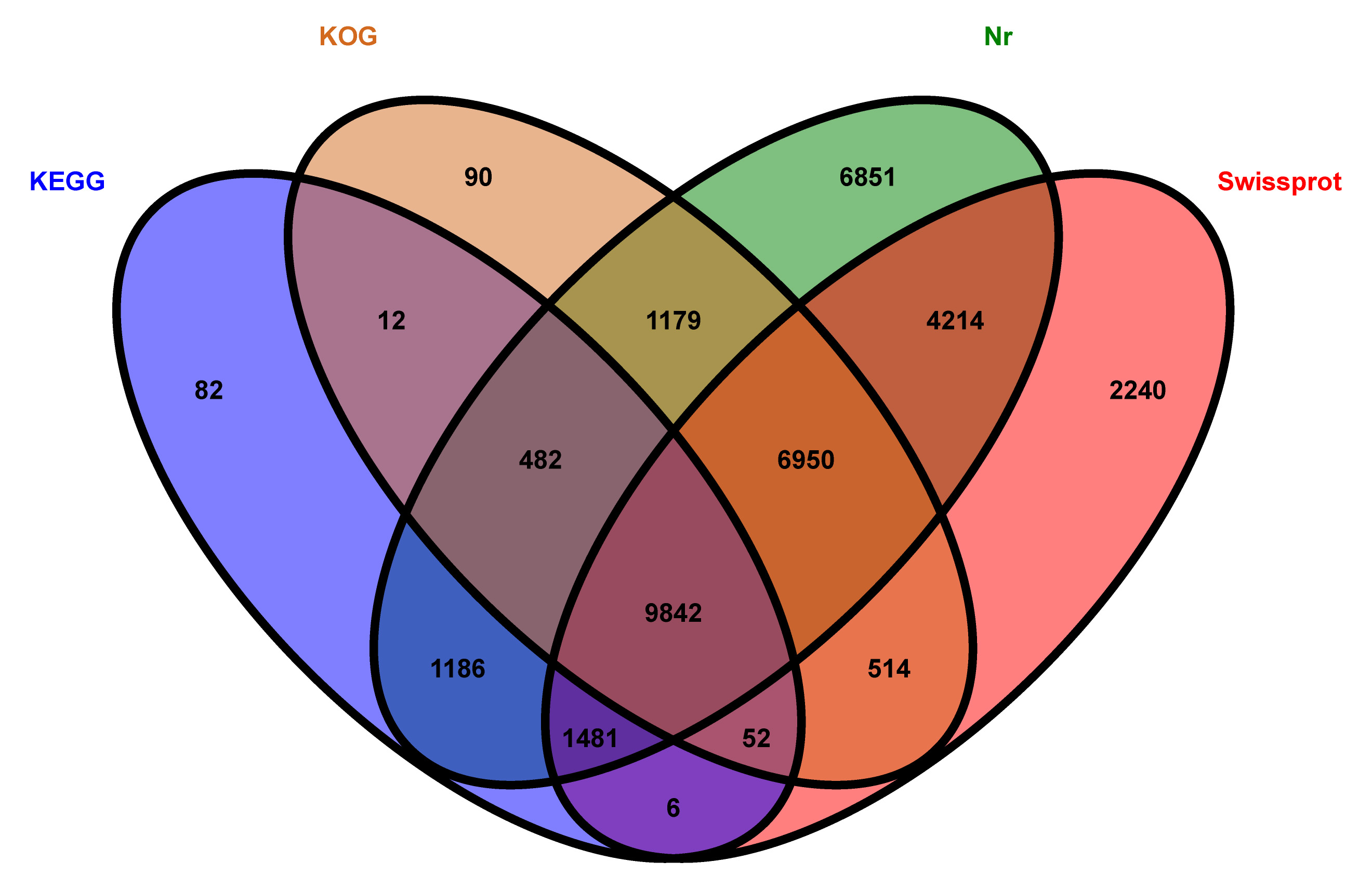

Supplement: Supplementary file 1 [file cimb-44-00434-s001.zip › Figure S1.jpg]

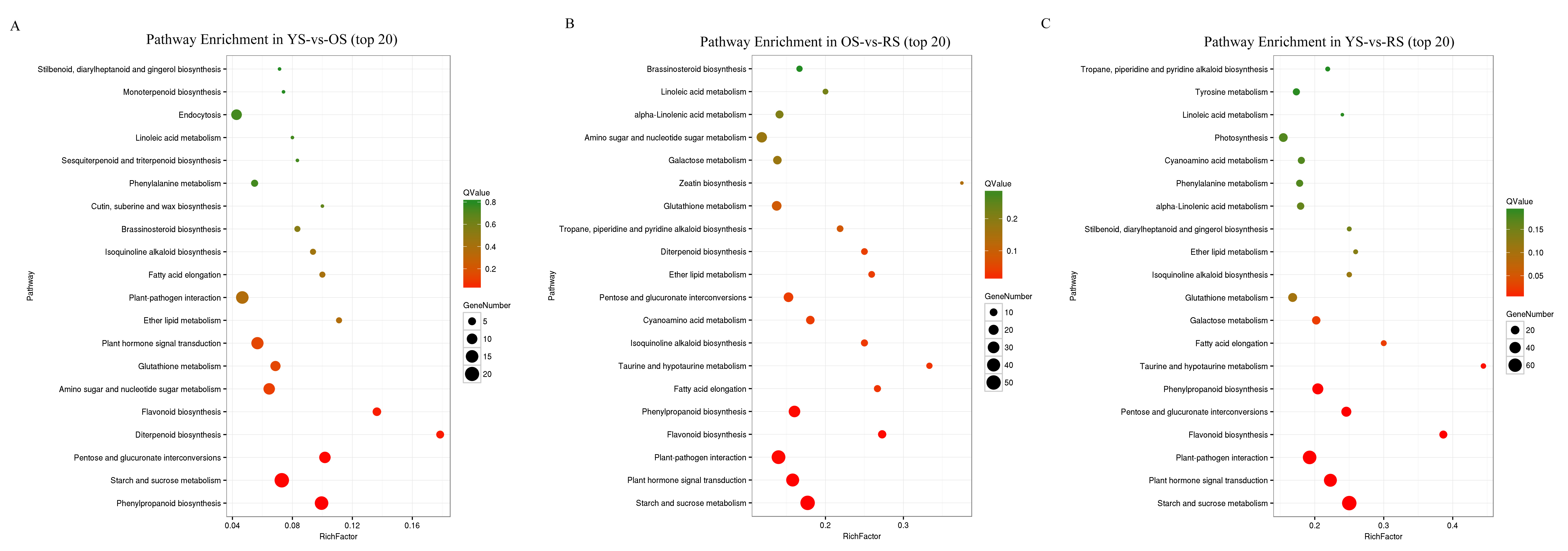

Supplement: Supplementary file 1 [file cimb-44-00434-s001.zip › Figure S2.jpg]

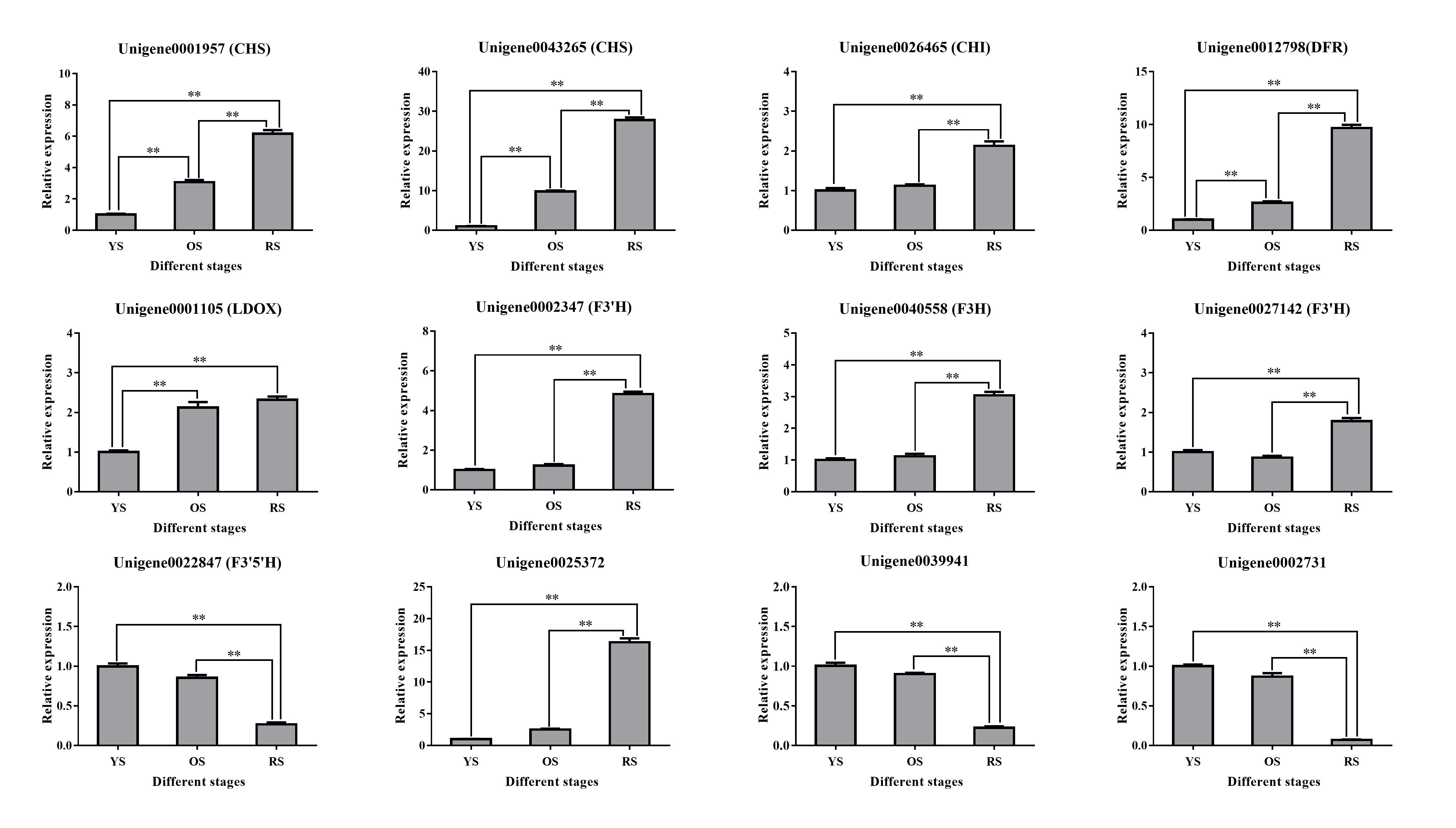

Supplement: Supplementary file 1 [file cimb-44-00434-s001.zip › Figure S3.jpg]
